# Molecular identification of tick-borne *Rickettsia*, *Anaplasma*, *Ehrlichia*, *Babesia*, and *Colpodella* in confiscated Malayan pangolins

**DOI:** 10.1371/journal.pntd.0012667

**Published:** 2024-11-22

**Authors:** Bing Li, Jun-Qiong Zhai, Ya-Jiang Wu, Fen Shan, Jie-Jian Zou, Fang-Hui Hou, Teng-Cheng Que, Wu Chen

**Affiliations:** 1 School of Basic Medical Sciences, Guangdong Provincial Key Laboratory of Pharmaceutical Bioactive Substances, Guangdong Pharmaceutical University, Guangzhou, China; 2 Guangzhou Zoo & Guangzhou Wildlife Research Center, Guangzhou, China; 3 Guangdong provincial wildlife rescue center, Guangzhou, China; 4 Faculty of Data Science, City University of Macau, Macau, China; Sri Balaji Vidyapeeth (Deemed to be University): Sri Balaji Vidyapeeth, INDIA

## Abstract

The traditional concept of “tonic food” and demand for traditional Chinese medicine make pangolins the largest population of illegally smuggled mammals in the world. Illegal hunting and trade are not only responsible for the sharp decline in pangolin populations but also provide conditions for pathogenic transmission. In 2021, we rescued 21 confiscated unhealthy Malayan pangolins, none of which survived. This study aimed to investigate the reasons for their unexpected deaths and the potential pathogens that may be transmitted during smuggling. Physical examination found that more than 80% pangolins were parasitized with *A*. *javanense* ticks. Autopsy and pathological staining analysis revealed multiple organ damage in the deceased pangolins. Pathogens nucleic acid detection of 33 tick samples showed that the positive rate of *Rickettsia* spp., *Anaplasma* spp., *Ehrlichia* spp. *Babesia* spp., and *Colpodella* spp. were 90.91%, 6.06%, 6.06%, 15.15% and 18.18%, respectively. Furthermore, pangolin samples were positive for *Rickettsia* spp. (42.86%, 9/21), *Ehrlichia* sp. (4.76%, 1/21), and *Babesia* sp. (4.76%, 1/21). This study confirmed that spotted fever triggered by *Rickettsia* spp. from *A*. *javanense* might accelerate the most death of confiscated pangolins, while *Ehrlichia* sp., and *Babesia* sp. infection potentially accelerating a few deaths. Of note, *A*. *javanense* ticks carrying *Colpodella* spp. were detected for the first time in Malayan pangolins. However, whether *Colpodella* spp. are pathogenic to pangolins is unknown. Further research on the diagnosis, treatment, surveillance, and elimination of ticks and tick-borne diseases in humans, livestock, and wildlife should provide insight into wildlife conservation and zoonotic disease prevention.

## Author summary

As a globally distributed obligate bloodthirsty ectoparasite, ticks are the vector second only to mosquitoes, capable of transmitting a variety of pathogenic viruses, bacteria, and protozoa to humans, livestock, and wildlife. Illegal poaching and smuggling pose a serious threat to the survival of pangolins and are also a potential route for the spread of tick-borne diseases. The present study was conducted to investigate the reasons for the unexpected death of Malayan pangolins confiscated in 2021, as well as to explore the potential pathogens that may be transmitted during smuggling. Based on the clinical symptoms, autopsy, and molecular detection results, we speculate that the primary cause was the spotted fever triggered by *Rickettsia* spp. from *A*. *javanense*, which accelerated the death of smuggled pangolins. *Ehrlichia* sp. or *Babesia* sp. infection might be associated with the death of a few pangolins. Moreover, *A*. *javanense* carrying *Colpodella* spp. were detected for the first time in Malayan pangolins, and whether *Colpodella* spp. are pathogenic to pangolins awaits further study. The findings suggest that more attention should be paid to the diagnosis and treatment of tick-borne diseases in the rescue operation of pangolins and other wildlife.

## Introduction

Pangolins are toothless mammals that feed on termites and are covered by distinctive overlapping keratinized scales. They have no natural predators, but their scales are highly valued in traditional Chinese medicine, making them one of the most illegally smuggled mammals in the world. Between August 2000 and July 2019, the international trafficking of pangolins and their derivatives involved an estimated 895 000 animals [1]. To strengthen their protection, the eight extant pangolin species were added to the International Union for Conservation of Nature (IUCN) Red List of threatened species in 2019 [2]. China also upgraded pangolins from a National Level II protected wild species to National Level I protected wild species in 2020. To increase numbers, many researchers have also attempted to initiate artificial breeding programs but have thus far been unsuccessful. Difficulties in captive breeding may be related to their specialized diets and poor stress responses, e.g., abnormalities in the immune, respiration, and digestive systems. In addition, the decrease in pangolin populations may be related to the occurrence of disease, with wild pangolins reported to be highly susceptible to infection by viruses, bacteria, and blood parasites [3].

Pangolins prey on insects and are also preyed upon by other wild animals, so protecting pangolins is crucial for maintaining food chain stability. Some countries in Asia and Africa have a tradition of hunting, consuming, and utilizing pangolins. Overhunting in Asia has led to a sharp decrease in the population of pangolins, which has also promoted illegal smuggling of African pangolins to Asia. Since the outbreak of COVID-19, the hidden dangers of the wildlife trade to public health security have attracted more considerable attention. Pangolins, bats, and other wildlife were considered as potential intermediate hosts of zoonotic pathogens such as Ebola virus, SARS-CoV-2, and HKU4-CoV-like viruses [4, 5], and other viruses which transmitted between wildlife and domestic animals, including paramyxovirus, astrovirus, pseudorabies virus, porcine circovirus 2 [5]. Therefore, illegal trade of pangolins not only destroys biodiversity and the environment, but also provides an important route for the transmission of new and recurrent infectious diseases.

Ticks are a diverse group of hematophagous ectoparasites found on mammals, birds, reptiles, and amphibians. While sucking blood, ticks also serve as pathogen vectors, transmitting viruses, bacteria, and protozoa, causing tick-borne diseases (TBDs) [6]. TBDs impose a significant threat to human healthy, livestock production, and wildlife survival, especially in temperate, tropical, and subtropical regions of the world [7,8]. Although analysis of ticks and TBDs in pangolins has deep significance for conservation and public health, relevant research remains limited. *Rickettsia africae*, the causative pathogen of African tick-bite fever, has been detected in *Amblyomma javanense* ticks on Malayan pangolins in Malaysia [9], Thailand [3], and southern China [10], and in *Amblyomma compressum* ticks on giant pangolins from the Congo [11]. *Haemaphysalis hystricis*, *Haemaphysalis formosensis*, and *Amblyomma testudinarium* have been reported in Formosan pangolins (*M*. *p*. *pentadactyla*) from Taiwan in China, with many of the *H*. *hystricis* ticks infected with *Rickettsia conorii* subsp., *Anaplasma* spp., *Ehrlichia* spp., and *Cytauxzoon* spp. [12]. *Candidatus Borrelia javanense* has been detected in *A*. *javanense* ticks on pangolins (*Manis javanica*) seized in anti-smuggling operations in southern China [13] and *Babesia* spp. have also been found in confiscated pangolins (*M*. *javanica*) in Thailand [14].

The present study was conducted to investigate the reasons for the unexpected death of confiscated Malayan pangolins and the potential pathogens that may be transmitted during smuggling. Our research not only provides a reference for the diagnosis of pangolin-related diseases and conservation but also for the prevention and control of zoonotic diseases.

## Materials and methods

### Ethics statement

This study was approved by the Guangzhou Zoo (Guangzhou Wildlife Research Center) Ethics Committee (approval number GZZOO2020031001). All procedures used during the research were in accordance with relevant guidelines and regulations.

### Pangolins

The 21 live Malayan pangolins studied here were rescued and treated by customs and the Department of Forestry of Guangdong Province in March 2021, then raised in a dark and quiet environment for further health assessment and rehabilitation by the Guangdong Provincial Wildlife Rescue Center at Guangzhou Zoo and the Guangdong Institute of Applied Biological Resources (China). Two mornings after the rescue, pangolins were anesthetized for physical examination and ectoparasites check, these operations complied with ethics approval (Wild Animal Treatment Regulation No. [2011] 85). All procedures used during the research were approved by the Guangzhou Zoo (Guangzhou Wildlife Research Center) Ethics Committee (approval number GZZOO2020031001).

### Tick collection and morphological identification

After careful physical examination, we found that 17 of the 21 pangolins were parasitized with ticks. The ticks were gently removed from the pangolins with tweezers and identified to species, life stage, and sex according to morphological criteria [15]. Finally, 16 male and 17 female ticks were stored at −80°C for DNA & RNA isolation and molecular detection.

### Pangolin tissue collection

Although active treatment methods were implemented, all 21 confiscated unhealthy pangolins ultimately died. Diagnostic necropsy was performed within six hours of all 21 pangolins death [16]. Carefully observed and recorded the gross changes on the body surface, external opening, and major organs. Tissues with typical lesions, including heart, lung, liver, kidney, spleen, were removed from pangolin carcass, which stored in 4% paraformaldehyde fix solution (Beyotime Biotechnology, Nantong, China) in sterile tubes and kept at room temperature, or directly stored in sterile tubes and kept at −80°C for further investigation, respectively.

### Hematoxylin and eosin (HE) staining

Tissues of dead pangolins stored in 4% paraformaldehyde fix solution at room temperature for at least 48 h were embedded in paraffin, cut into sections of 5 μm in thickness and sticked onto the glass slide. After deparaffinized and rehydrated, the sections on slides were stained with H&E staining kit (Abcam, Cambridge, UK) according to the guidance, mounted with coverslips and observed using a suit of Olympus equipment (BX53 with PM-C 35 digital camera).

### Nucleic acid extraction of ticks and pangolin samples

Tick or pangolins tissues homogenates were prepared using a frozen tissue homogenizer (SCIENTZ, Ningbo, China) after adding twice the volume of phosphate-buffered saline (PBS). The homogenate or pangolins blood DNA and RNA were extracted using Tissue/Blood DNA/RNA Kit (OMEGA, Norcross, Georgia, USA) as described by the manufacturer respectively. Extracted DNA and RNA were stored at ˗80°C for further pathogens detection.

### Molecular detection of ticks and pathogens

The total RNA of tick samples was used as template for species identified by the species primer pairs (Table 1) targeted 16S rRNA [17]. The RT-PCR reaction was conducted using the TaKaRa PrimeScript One Step RT-PCR Kit (TaKaRa, Shiga, Japan) according to the product information.

For the detection of RNA viral pathogens including avian influenza virus (AIV), coronavirus (CoVs), canine distemper virus (CDV), encephalomyocarditis virus (ECMV), parainfluenza virus type 5 (PIV5). The total RNA of ticks, pangolins tissue or blood samples was used as template for RT-PCR reaction and specifically amplified using primers as described above. The DNA extracted from ticks or pangolins samples was used as template for DNA viral pathogens detection (canine herpes virus (CHV) and canine parvovirus (CPV)), procaryotic pathogens detection (*Rickettsia*, *Ehrlichia* and *Anaplasma*) [18], and protozoon pathogens detection (*Babesia*, *Theileria* and *Hepatozoon*) [19]. Primers used in this study were listed in Table 1.

**Table 1:** Primers used in this study.

Primers	Corresponding sequences (5’to3’)	Reference
Tick-16S–F	CTGCTCAATGATTTTTTAAATTGCTGTGG	[17]
Tick-16S–R	CCGGTCTGAACTCAGATCAAGT	
AIV-F	ATGAGYCTTCTAACCGAGG	This study
AIV-R	CGTCTACGCTGCAGTCCT	
CDV-F	ATAGATGTCTTGACACCGCTCTT	
CDV-R	GTACATACCTTGGCTTTGGAACT	
CHV–F	GGTAGACCCTCCTCGTAGGTAT	
CHV–R	GGGGCAGCTAAAACTAATCCCA	
Pan-CoVs-OF1	TGTTATTGGAACAACTAAATTYTYGGIGGITG	
Pan-CoVs-OR1	GGTTGCATCACCACTACTAGTICCNCCIGGYTT	
Pan-CoVs-OF2	GTTTTGAAAATCCTATTCTTATGGGITGGGAYTAYCC	
Pan-CoVs-OR2	GGTTGCATCACCACTACTAGTICCNCCIGGYTT	
CPV-F	GTAAGCTTCCAGGAGACTTT	
CPV-R	GTAAGCTTCGTCGTGTTCTT	
EMCV-F	TCTGTTGAATGTCGTGAAGGA	
EMCV-R	AGGCCCCAGATCAGATCC	
Rickettsiaceae17KDa 1st-F	TTTACAAAATTCTAAAAACCAT	[18]
Rickettsiaceae17KDa 1st-R	TCAATTCACAACTTGCCATT	
Rickettsiaceae17KDa 2nd-F	GCTCTTGCAACTTCTATGTT	
Rickettsiaceae17KDa 2nd-R	TCAATTCACAACTTGCCATT	
Anaplasmataceae16S 1st-F	GAACGAACGCTGGCGGCAAGC	
Anaplasmataceae16S 1st-R	CACGCTTTCGCACCTCAGTGTC	
Anaplasmataceae16S 2nd-F	TGCRTAGGAATCTRCCTAGTAG	
Anaplasmataceae16S 2nd-R	CACGCTTTCGCACCTCAGTGTC	
BTH 18S 1st-F	GTGAAACTGCGAATGGCTCATTAC	[19]
BTH 18S 1st-R	AAGTGATAAGGTTCACAAAACTTCCC	
BTH 18S 2nd-F	GGCTCATTACAACAGTTATAGTTTATTTG	
BTH 18S 2nd-R	CGGTCCGAATAATTCACCGGAT	

The PCR products were run in 3% agarose gel (Thermo Fisher Scientific, Waltham, MA, US) with FluoroVue Nucleic Acid Gel Stain (SMOBIO, Beijing. China) and electrophoresed at 160 V for 25 min to separate DNA fragments. Nucleic acid bands were visualized under ultraviolet (UV) light, and positive bands were excised and purified with a TIANamp Genomic DNA Kit (Tiangen, Beijing, China) and sequenced (Ruibiotech, Beijing, China). The obtained nucleotide sequences were compared with sequences published in GenBank using BLAST (https://blast.ncbi.nlm.nih.gov/Blast.cgi) to search for sequence homology.

### Phylogenetic tree analysis

Phylogenetic analysis of the PCR-obtained sequences of ticks were conducted using MEGA v6.0 with the maximum-likelihood algorithm. Bootstrap values were calculated with 1 000 replicates.

## Results

### Observations and clinical symptoms of confiscated Malayan pangolins

Upon rescue, all 21 pangolins including four underage female (weight in 1.95 kg to 3.2 kg), ten adult females (weight in 4.0 kg to 5.7 kg) and seven adult males (weight in 7.5 kg to 8.3 kg), exhibited poor vigor, accompanied by various symptoms such as cough, drowsiness, anorexia, and edema of extremities. Rescuers also observed inexplicable wounds on the body surface of pangolins, which may have been caused by hunting or transportation. In total, 17 of the 21 pangolins (80.95%) were parasitized with ticks, and some tick bite wounds were broken and infected. After gently removing all ticks, the wounds were disinfected with normal saline and compound lysozyme disinfectant spray (Kalo, Kunshan, China). Intramuscular injection of Synulox RTU (Zoetis, New York, USA) (0.05 ml/kg body weight) once daily for three consecutive days. Although active antibiotic treatment methods were implemented, clinical symptoms were not relieved, some tick bite and wounds had not recovered, and the pangolins died one after another from an unknown cause. Before death, several pangolins also exhibited bloody stools, hematuria, convulsions, and other neurological symptoms.

### Identification and phylogenetic analysis of ticks

**Fig 1:**
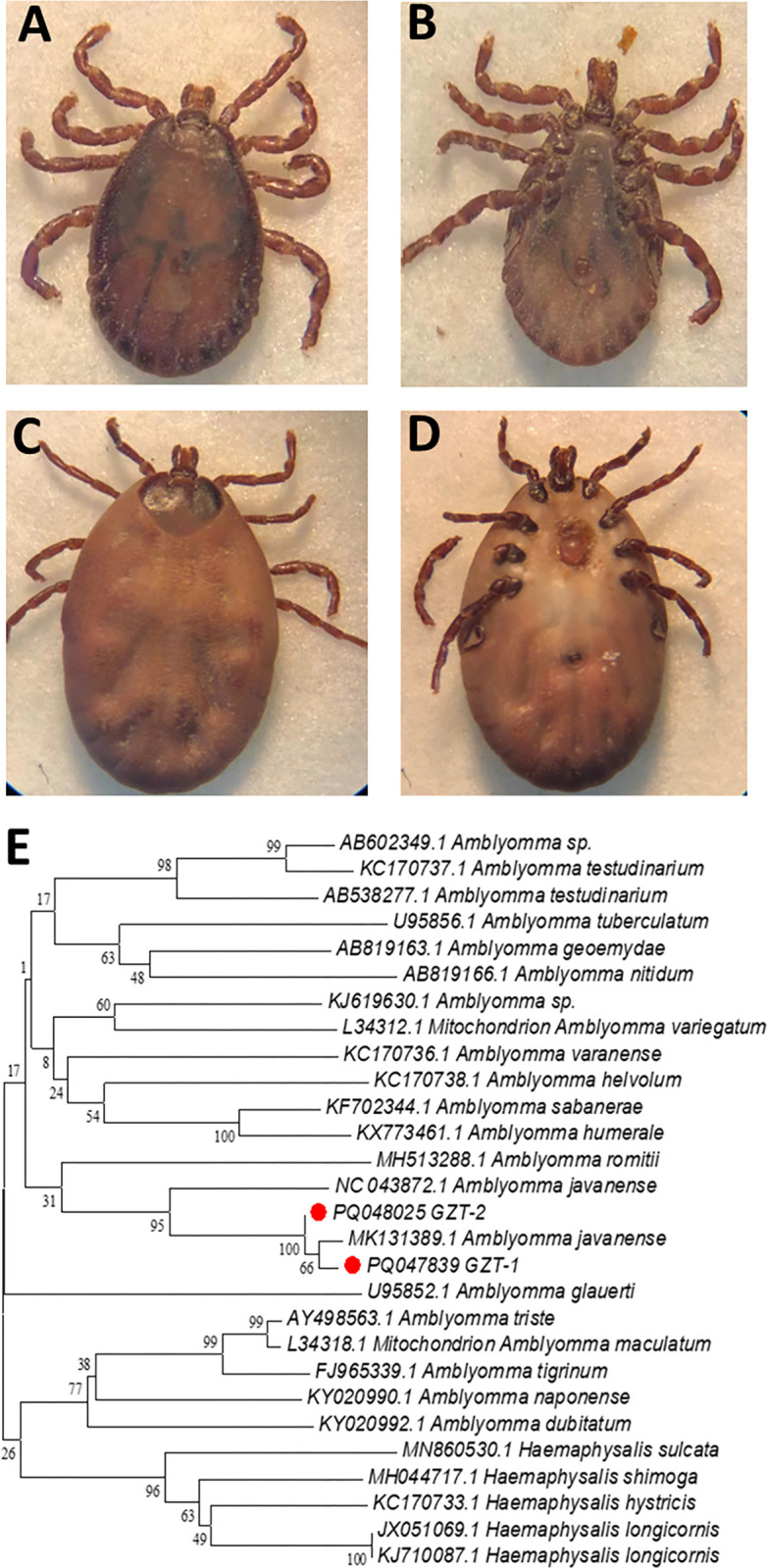
Morphologic and molecular identification of ticks on the surface of confiscated Malayan pangolins. **(**A)&(B) Dorsa view and ventral view of male *A*. *javanense* tick. **(**C)&(D) Dorsa view and ventral view of female *A*. *javanense* tick. (E) Phylogenetic tree based on the 16S rRNA of ticks from confiscated Malayan pangolins. Analyses were conducted using MEGA v6.0 with the maximum-likelihood algorithm. Bootstrap values were calculated with 1000 replicates. The number on each branch indicates bootstrap value. Red circle: sequences of ticks obtained in this study.

Based on the morphological characteristics [15], ticks collected from the confiscated pangolins were initially identified as *Amblyomma javanense* (*A*. *javanense*) (Fig 1A–1D). To further confirm the tick species, specific DNA sequences were amplified from cDNA of two ticks and sequenced for 16S rRNA. BLAST results showed that 16S rRNA gene of ticks (Genbank PQ047839 and PQ048025) exhibit 99% similarity with *A*. *javanense*. Genetic evolution showed that the selected sequences of 16S rRNA gene belonged to the same branch as *A*. *javanense* (Fig 1E).

### Autopsy, microscopic lesion and blood tests of dead Malayan pangolins

**Fig 2:**
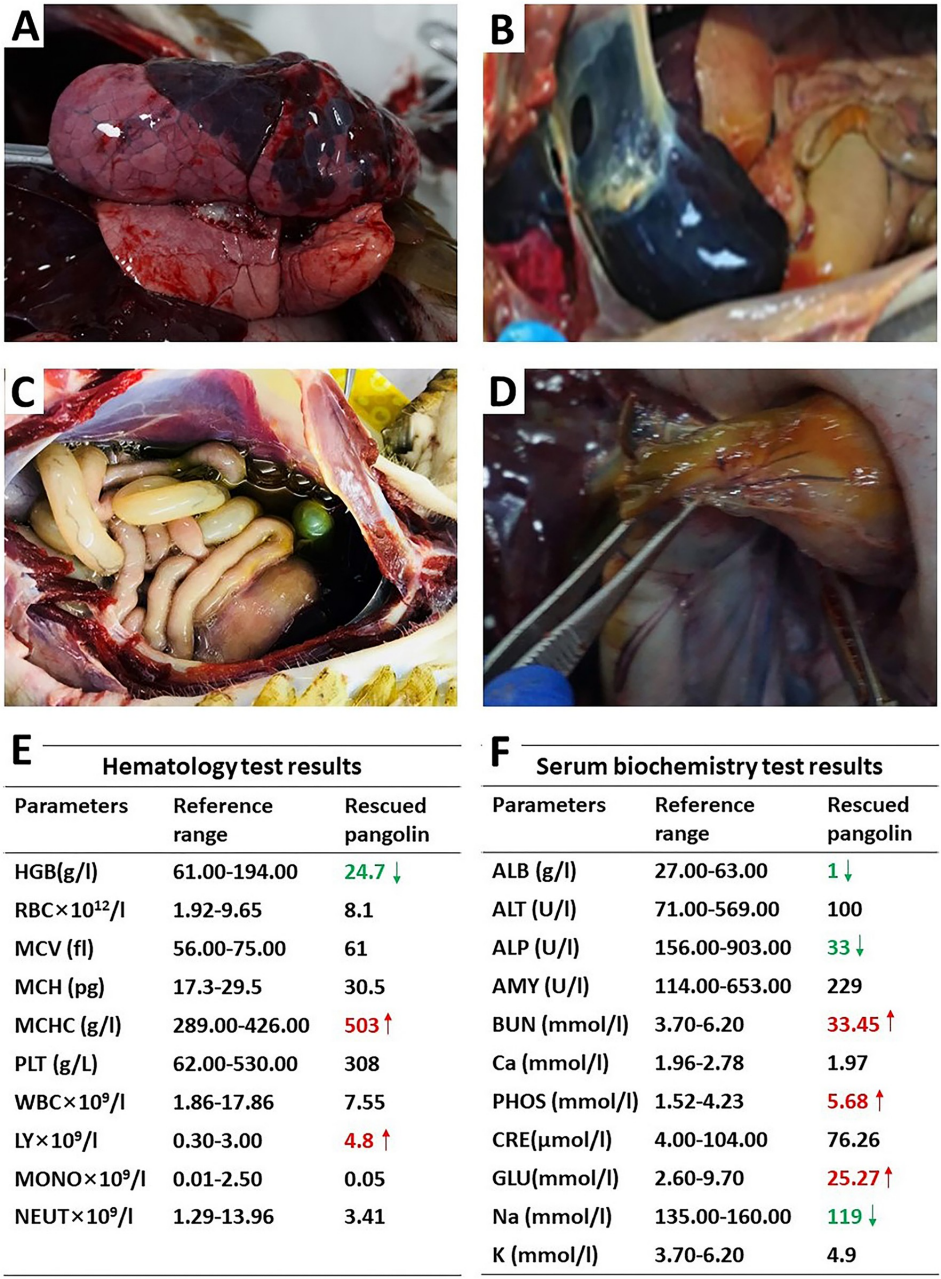
Gross pathological lesions of dead Malayan pangolins after autopsy. **(**A**)** Congestion and hemorrhage observed in the lung. (B) Myocardial edema, myocardial collapse, and ventricular congestion were evident in the heart. (C) obvious ascites, intestinal bloating. (D) serosa of gastric and duodenal turned yellow. (E)&(F) blood testing showed levels of HGB (hemoglobin), MCHC (mean corpuscular hemoglobin concentration), LY (lymphocyte), ALB (albumin), BUN (blood urea nitrogen), PHOS (phosphate), GLU (glucose), and Na (Natrium) were out of the reference range.

In the autopsy of pangolins, congestion and edema were observed in most internal organs, especially lung and heart (Fig 2A and 2B). Ascites, flatulence and yellow serous membrane of digestive tract were shown in some pangolins (Fig 2C and 2D). Large hemorrhage region, spots of hemorrhage were observed on the surface of the lung (Fig 2A). Additionally, the cut surface of the lungs showed infiltration with foamy fluid. Myocardial edema, myocardial collapse, and ventricular congestion were evident in the heart. (Fig 2B). Some pangolins showed obvious ascites, intestinal bloating (Fig 2C), and the serosa of gastric and duodenal turned yellow (Fig 2D). The results of the hematologic and serum biochemical test also revealed that pangolins had mostly anemic appearance, inflammation and hepatorenal dysfunction (Fig 2E and 2F).

**Fig 3:**
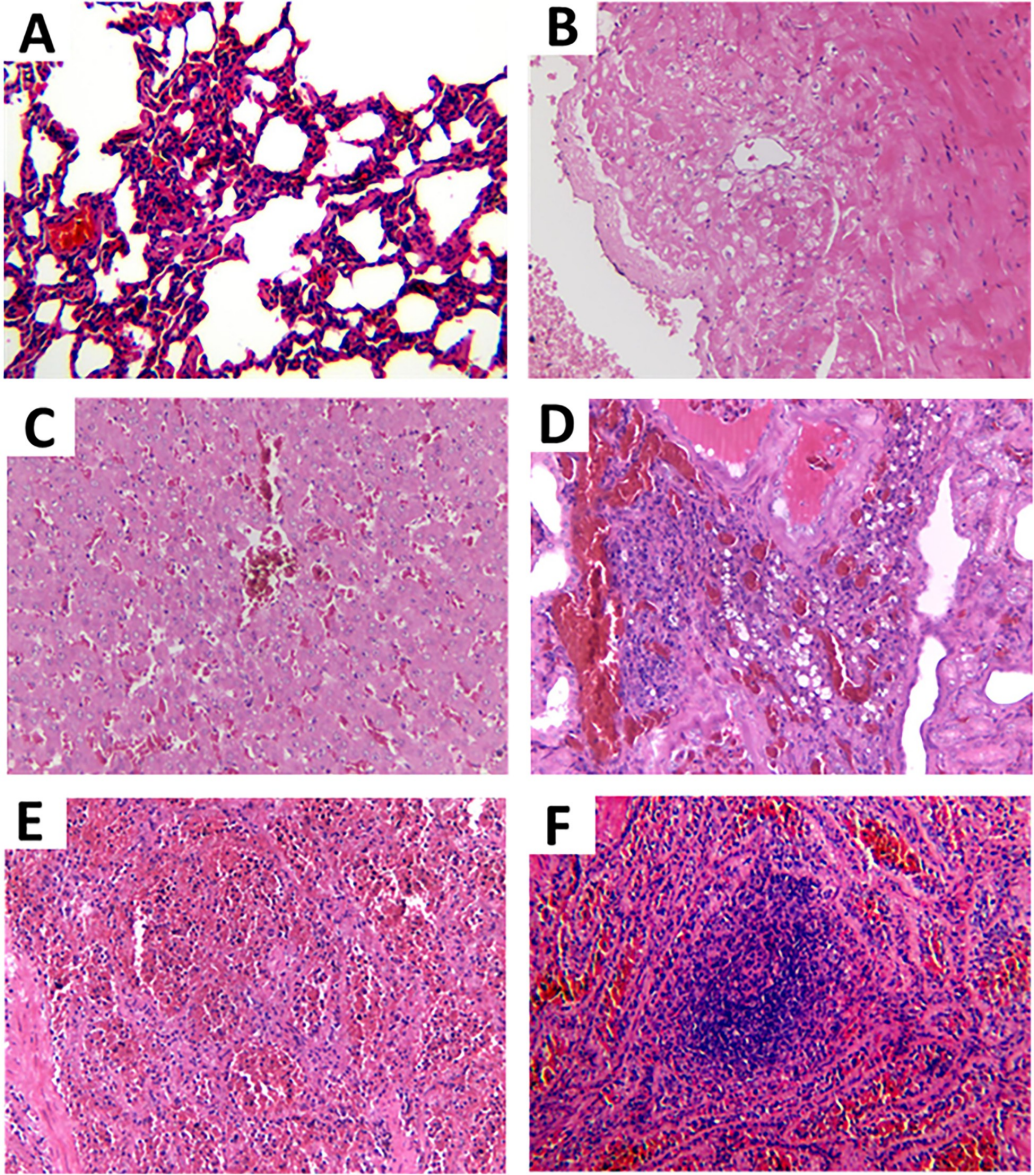
Pathological changes of dead Malayan pangolins. (A) Lung, alveolar collapse, alveolar wall thickening, inflammatory cell infiltration, and capillary dilation and congestion. (B) Heart, myocardial fibers vacuolar denaturation. (C) Liver, hepatic sinusoid, hepatic cytoplasmic vacuolization and hepatocyte edema. (D) Kidney, the glomerulus capillary is expandable and hyperemia. Renal tubular epithelial cells exhibit edema, vacuolar degeneration, dilation, and calcification. Fibrous tissue hyperplasia, capillary dilation, and inflammatory cell infiltration are observed in renal interstitial. (E) Spleen, splenic congestion with hemosiderin deposition in the red pulp. (F) Lymph nodes, the thin cortex, absence of lymph nodes, medullary vascular dilation and congestion, and numerous macrophages observed in the spinal cord.

Histopathological analysis revealed a significant presence of inflammation across multiple organs, including lung, heart, liver, kidney, spleen and Lymph nodes (Fig 3). The microscopic lesions included alveolar collapse, alveolar wall thickening, inflammatory cell infiltration, and capillary dilation and congestion (Fig 3A); myocardial fibers vacuolar denaturation (Fig 3B); hepatic sinus congestion, hepatic cytoplasmic vacuolization and hepatocyte edema (Fig 3C); the glomerulus capillary is expandable and hyperemia; renal tubular epithelial cells exhibit edema, vacuolar degeneration, dilation, and calcification; fibrous tissue hyperplasia, capillary dilation, and inflammatory cell infiltration were observed in renal interstitial (Fig 3D); splenic congestion with hemosiderin deposition in the red pulp (Fig 3E); the thin cortex, absence of lymph nodes, medullary vascular dilation and congestion, and numerous macrophages observed in the spinal cord (Fig 3F). To sum up, the confiscated Malayan pangolins were more likely to die of Multiple Systems Organ Failure.

### Identification and phylogenetic analysis of tick-borne pathogens

Ticks, tissues and blood samples from the confiscated pangolins were collected for pathogens detection. The results of PCR and RT-PCR showed that all samples were negative for viral pathogens (AIV, CDV, CHV, CPV, CoVs, EMCV and PIV5). Furthermore, the DNA samples were amplified to detect *Rickettsia*, *Anaplasma*, *Babesia*, *Theileria*, and *Hepatozoon* species (Table 2).

**Table 2:** Tick-borne pathogens detected by PCR in ticks and tissues collected from Malayan pangolins.

Pathogen	Pangolins		Ticks	
	No. of Pangolins: tissue	Infected rate (%)	No. of Ticks	Infected rate (%)
*Rickettsiaceae*	Pangolin1: blood Pangolin2: lung Pangolin3: blood Pangolin4: spleen Pangolin6: blood Pangolin12: lung Pangolin14: blood Pangolin16: blood Pangolin20: spleen	9/21 (42.86)	PT1-1♂, PT1-2♀ PT2♀ PT3-1♂, PT3-2♀ PT4♂ PT5-1♂, PT5-2♀ PT6♀ PT8♀ PT9-1♂, PT9-2♀ PT11-1♂, PT11-2♀ PT12-1♂, PT12-2♂ PT14-2♀ PT16-1♂, PT16-2♂, PT16-3♂, PT16-4♀, PT16-5♂, PT16-6♀ PT17-1♀, PT17-2♀ PT18-1♂ PT19-1♂, PT19-2♀ PT20-1♂, PT30/20-2♀	33/30 (90.91)
*Anaplasmataceae*	Pangolin12: lung	1/21 (4.76)	PT1-2♀ PT12-1♂ PT17-1♀, PT17-2♀	4/33 (12.12)
*Babesia/ Theileria/ Hepatozoon*	Pangolin3: blood	1/21 (4.76)	PT1-2♀ PT2♀ PT3-1♂ PT4♂ PT9-1♂ PT11-2♂ PT12-1♂ PT14-2♀ PT16-2♂ PT17-1♀ PT18-2♀	11/33 (33.33)

CM: ♀: Female; ♂: Male; Underscore: Double infections; Gray background: Triple infections.

#### Rickettsiaceae

*Rickettsia* sp. was the most common tick-borne pathogen identified in the tick samples and pangolin samples, accounting for 90.91% (30/33) and 42.86% (9/21) respectively. A homology search of the generated sequences (~410 bp) revealed that the sequences of the *Rickettsiaceae* 17 kDa gene amplified from ticks and pangolins had 77.23%–100.00% similarity to the *Rickettsia* sequences in GenBank. By comparing sequences, we found considerable differences in homology among sequences (75.50% to 100.00%). Sequences from the tick samples (PT1-2, PT2, PT3-2, PT4, PT6, PT8, PT9-1, PT12-1, PT12-2, PT16-2, and PT20-2) and the pangolin samples (Pangolin1, Pangolin3, Pangolin4, Pangolin6, Pangolin9, Pangolin12, and Pangolin20), showed 96.88%–100.00% identity with *Candidatus Rickettsia* (GenBank MH932031 and MH932038) (Table 3). Sequences from the tick samples (PT14-2, PT16-4, and PT16-6) and pangolin samples (Pangolin14 and Pangolin16) exhibited 98.49%, 99.03%, 99.49%, 98.52% and 98.75% similarity, respectively, with *Rickettsia rhipicephali* (GenBank CP003342) (Table 3). Sequences from the PT5-1, PT17-1, and PT20-1 samples showed 87.13% similarity with *Rickettsia rickettsii* (GenBank CP018914), 82.37% similarity with *Rickettsia japonica* (CP047359), and 86.63% similarity with unclassified *Rickettsia* (GenBank KT261767), respectively (Table 3). The PT1-1, PT3-1, PT5-2, PT9-2, PT11-1, PT11-2, and PT17-2 samples also showed 79.08%–79.95% similarity with uncultured *Rickettsia* sp. (GenBank GQ302893) (Table 3). The remaining sequences amplified from the PT16-1, PT16-3, PT16-5, PT18-1, PT19-1, and PT19-2 samples exhibited 77.23%–78.90% homology to *Rickettsia rhipicephali* (GenBank CP013133) (Table 3).

**Table 3:** GenBank Blast search results showing percent identity of the various *Rickettsia*, *Babesia*, *Ehrlichia*, *Colpodella* and *Anaplasma* identified in ticks.

Bacterial species (Genbank Species ID)		Tick			Pangolin		
		No. of ticks	GenBank accession numbers	Percent identity (%)	No. of pangolins:	GenBank accession numbers	Percent identity (%)
***Rickettsia***	*Candidatus Rickettsia jingxinensis* (MH932038)	PT1-2	PQ133367	99.50	Pangolin1	PQ133378	99.01
		PT2	PQ133368	99.50	Pangolin2	PQ133379	100.00
		PT20-2	PQ133369	100.00	Pangolin20	PQ133386	99.47
	*Candidatus Rickettsia jingxinensis* (MH932031)	PT3-2	PQ133370	99.28	Pangolin3	PQ133380	99.28
		PT4	PQ133371	100.00	Pangolin4	PQ133381	99.50
		PT6	PQ133372	99.27	Pangolin6	PQ133382	98.53
		PT8	PQ133373	99.75	Pangolin12	PQ133383	99.49
		PT9-1	PQ133374	99.49			
		PT12-1	PQ133375	100.00			
		PT12-2	PQ133376	96.64			
		PT16-2	PQ133377	99.75			
	Uncultured *Rickettsia* sp. (GQ302893)	PT1-1	PQ133387	79.95			
		PT3-1	PQ133388	78.32			
		PT5-2	PQ133389	80.36			
		PT9-2	PQ133390	79.39			
		PT11-1	PQ133391	79.47			
		PT11-2	PQ133392	79.89			
	Uncultured *Rickettsia* sp. (KT261767)	PT20-1	PQ133402	86.32			
	*Rickettsia rickettsii* (CP018914)	PT5-1	PQ133400	87.09			
	*Rickettsia rhipicephali* (CP003342)	PT14-2	PQ133364	98.49	Pangolin14	PQ133384	98.52
		PT16-4	PQ133365	99.03	Pangolin16	PQ133385	98.75
		PT16-6	PQ133366	99.49			
	*Rickettsia rhipicephali* (CP013133)	PT16-1	PQ133393	77.23			
		PT16-3	PQ133394	78.30			
		PT16-5	PQ133395	78.90			
		PT17-2	PQ133396	78.06			
		PT18-1	PQ133397	78.36			
		PT19-1	PQ133398	78.30			
		PT19-2	PQ133399	78.30			
	*Rickettsia japonica* (CP047359)	PT17-1	PQ133401	82.37			
***Ehrlichia***	*Ehrlichia ruminantium* (NR_074155)	PT1-2:	PQ056502	99.65	Pangolin12	PQ136550	99.46
		PT12-1	PQ056503	99.64			
***Anaplasma***	Uncultured *Anaplasma* sp. (KU189193)	PT17-1	PQ056505	99.30			
		PT17-2	PQ056506	99.65			
***Babesia***	*Babesia* sp. (MT256300)	PT1-2	PQ048968	95.93	Pangolin3	PQ136913	95.66
		PT2	PQ049133	96.00			
		PT3-1	PQ049142	95.96			
		PT9-1	PQ049597	95.91			
		PT14-2	PQ04960	94.10			
***Colpodella***	*Colpodella* sp. (MH012046)	PT4	PQ049327	100.00			
	*Colpodella* sp. (KT600661)	PT11-2	PQ049601	95.55			
		PT12-1	PQ049602	98.99			
		PT16-2	PQ049604	95.40			
		PT18-2	PQ056103	94.38			
	*Colpodella* sp. (MH208621)	PT17-1	PQ056084	99.78			

**Fig 4:**
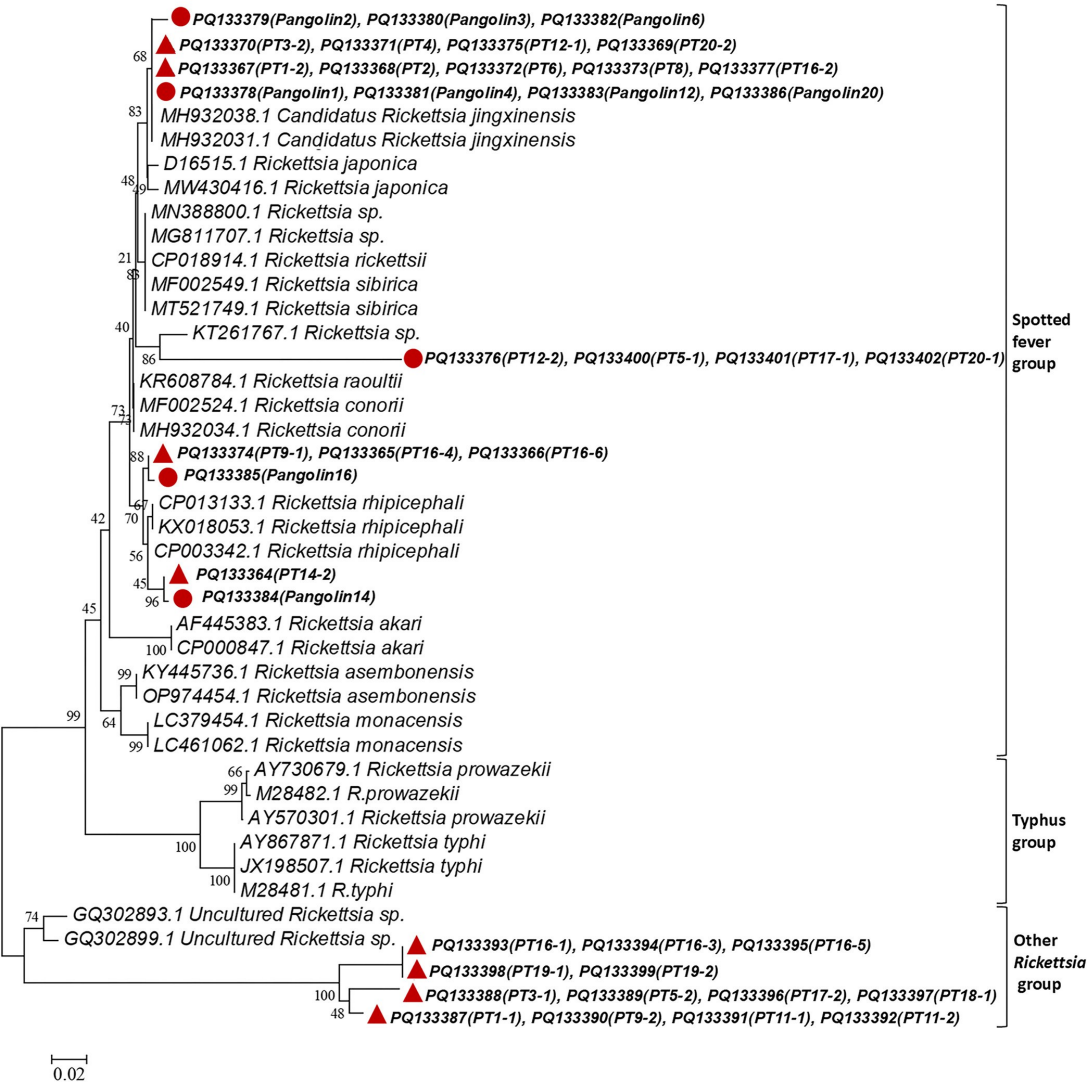
Phylogenetic tree based on 17 kDa gene fragment of *Rickettsiaceae* found in ticks on confiscated pangolins. Analyses were conducted by using MEGA v6.0 with maximum-likelihood algorithm. Bootstrap values were calculated with 1 000 replicates. Number on each branch indicates bootstrap values. Red circle: sequences amplified from pangolin samples in this study. Red triangle: sequences amplified from tick samples in this study.

The phylogenetic trees demonstrated that sequences from the tick samples (PT20-2, PT16-2, PT12-1, PT8, PT6, 4, 3–2, 2, and 1–2) and pangolin samples (Pangolin1, Pangolin2, Pangolin3, Pangolin4, Pangolin6, Pangolin12, and Pangolin20) were in the same clade as *Candidatus Rickettsia* (GenBank MH932031 and MH932038). Sequences from the PT14-2, PT16-4, PT9-1, PT16-6, Pangolin16, PT14-1, and Pangolin14 samples were close to *Rickettsia rhipicephali* (GenBank CP013133). Sequences from the PT12-2, PT5-1, PT17-1, and PT20-1 samples appeared in the same clade as, but distinct from, *Rickettsia* sp. (Genbank KT261767). Sequences from the PT1-1, PT9-2, PT11-1, PT11-2, PT3-1, PT5-2, PT17-2, PT18-1, PT16-1, PT16-3, PT16-5, PT19-1, PT19-2, samples were clustered in the same clade as, but distinct from, uncultured *Rickettsia* sp. (GenBank GQ302893 and GQ302899) (Fig 4). Significantly, the phylogenetic trees demonstrated that the sequences from the pangolin samples all closely resembled spotted fever group (SFG) members.

#### Anaplasmataceae

**Fig 5:**
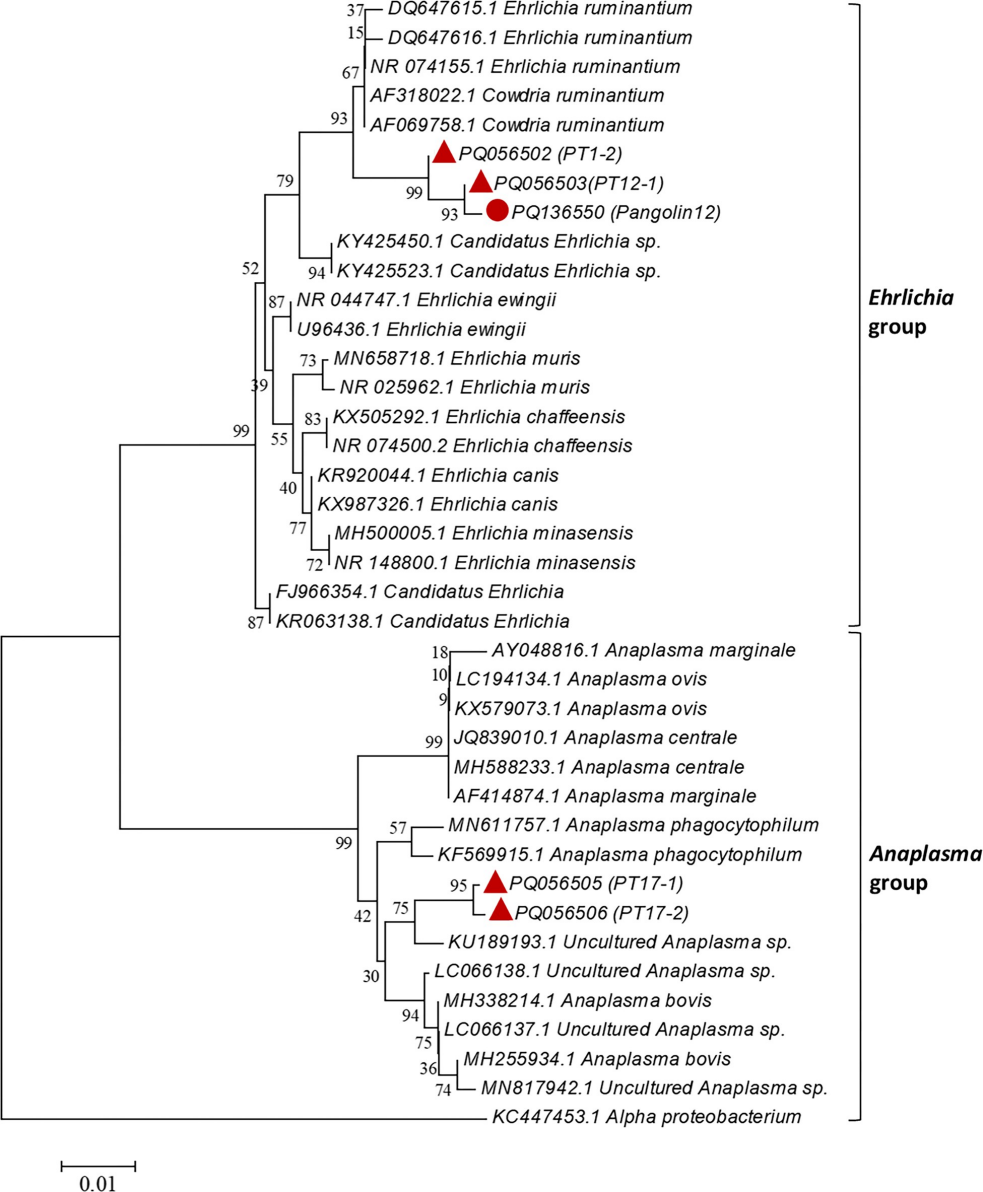
Phylogenetic tree based on 16S rRNA gene fragment of *Anaplasmataceae* found in ticks on confiscated pangolins. Analyses were conducted using MEGA v6.0 with maximum-likelihood algorithm. Bootstrap values were calculated with 1 000 replicates. Number on each branch indicates bootstrap values. Red circle: sequence amplified from pangolin sample in this study. Red triangle: sequences amplified from tick samples in this study.

A 1 458 bp fragment of the 16S rRNA gene of *Anaplasmataceae* was detected in 12.12% (4/33) of tick samples and 4.76% (1/21) of pangolin samples. Based on phylogenetic analysis, the sequences amplified from the PT1-2, PT12-1 and Pangolin12 showed 99.65%, 99.64% and 99.46 similarity, respectively, to *Ehrlichia ruminantium* (GenBank NR_074155.1) and were located in the same clade (Table 3 and Fig 5). Sequences from the PT17-1 and PT17-2 samples showed 99.30% and 99.65% similarity, respectively, to uncultured *Anaplasma* sp. (GenBank KU189193.1) and were also located in the same clade (Table 3 and Fig 5). Therefore, the positivity rate of *Ehrlichia* spp. and *Anaplasma* spp. of tick samples were both 6.06%, and the pangolin samples was only positive for *Ehrlichia* spp., accounting for 4.76% (1/21).

#### Babesia, Theileria, and Hepatozoon

**Fig 6:**
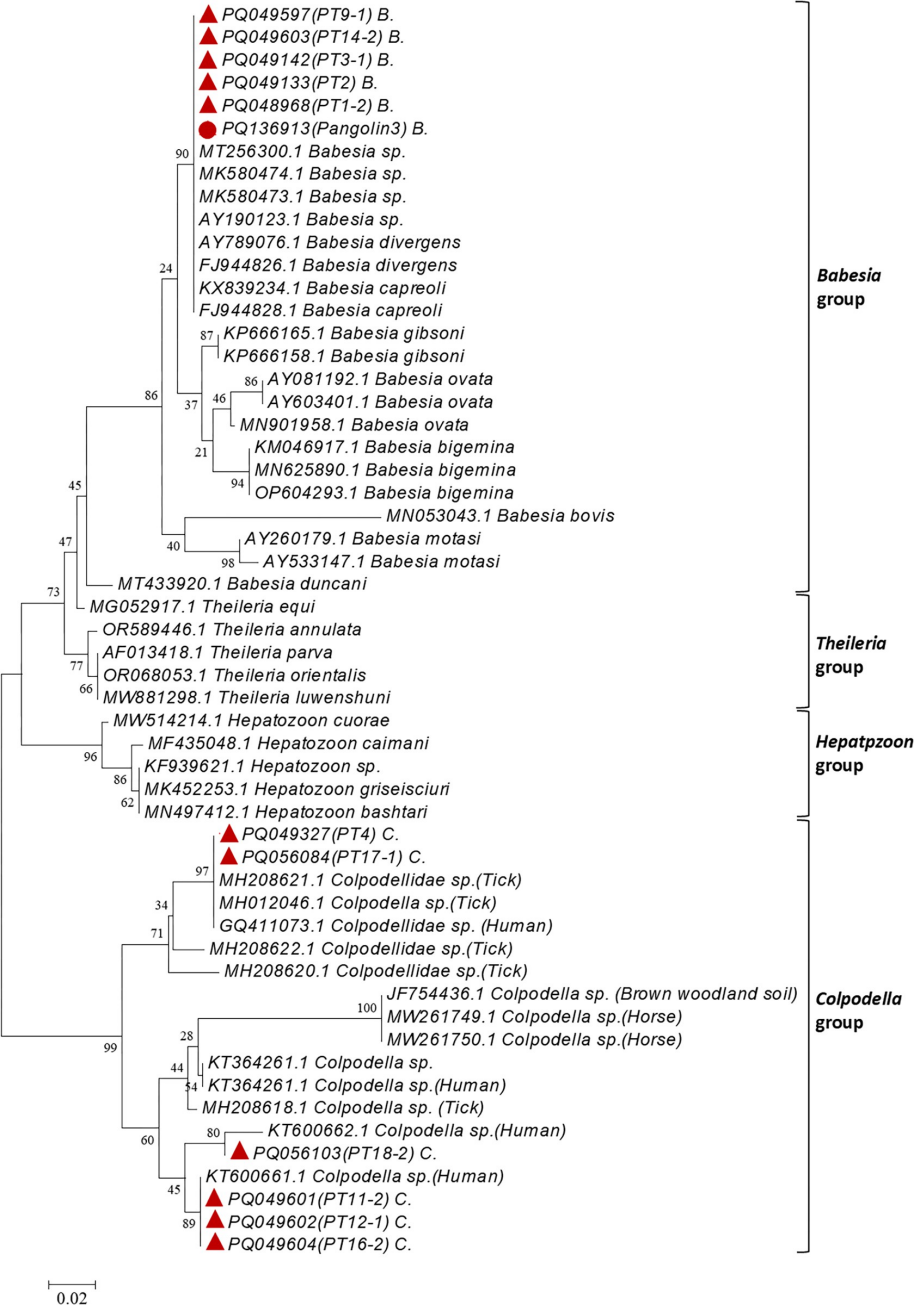
Phylogenetic tree based on nucleotide sequences of 18S rRNA genes of *Babesia* spp. and *Colpodella* spp. found in ticks on confiscated pangolins. Analyses were conducted using MEGA v6.0 with maximum-likelihood algorithm. Bootstrap values were calculated with 1 000 replicates. Number on each branch indicates bootstrap values. Red circle: sequence amplified from pangolin sample in this study. Red triangle: sequences amplified from tick samples in this study.

The positive rate of the BTH primer in tick and pangolin samples were 33.33% (11/33) and 4.76% (1/21), respectively. Sequences (~1 604 bp) of PT1-2, PT2, PT3-1, PT9-1, PT14-2 and Pangolin3 exhibited 94.10%–96.00% similarity to *Babesia* sp. (GenBank MT256300) and were clustered in the same clade (Table 3 and Fig 6). Sequences of the PT11-1, PT12-1, PT16-2, and PT18-2 samples showed 94.38%–98.99% similarity to *Colpodella* sp. (GenBank KT600661) and were clustered in the same clade. The PT4 and PT17-1 samples exhibited 100.00% and 99.78% similarity, respectively, to *Colpodella* sp. (GenBank GQ411073) and were also found in the same clade (Table 3 and Fig 6). Based on the above analysis, the positive rate of *Babesia* spp. in tick samples was 15.15% (5/33), that of C*olpodella* spp. was 18.18% (6/33). However, only *Babesia* spp. was detected in pangolin samples, with a positive rate of 4.76% (1/21). Neither *Theileria* sp. nor *Hepatozoon* sp. was detected in tick and pangolin samples.

#### Analysis of co-infection

co-infection of ticks was common in this study (Table 2), including single ticks carrying multiple pathogens and different ticks on the same pangolin carrying different pathogens. Multiple infections were found in 33.33% (11/33) of samples. *Rickettsia* sp. (30/33; 90.91%) was most frequently associated with multiple infection, followed by *Babesia* sp. (5/33;15.15%). Double infections accounted for 24.24% (8/33) of samples, with *Rickettsia* sp. + *Babesia* sp. co-infection showing the highest overall prevalence (12.12%; 4/33), followed by *Rickettsia* sp. + *Colpodella* sp. (9.09%; 3/33), and *Rickettsia* sp. + *Anaplasma* sp. (3.03%; 1/33). Triple infections also occurred in several ticks, including *Rickettsia* sp. + *Ehrlichia* sp. + *Babesia* sp. (3.03%; 1/33) and *Rickettsia* sp. + *Ehrlichia* sp. + *Colpodella* sp. (6.06%; 2/33). In pangolins, 9 cases (42.86%, 9/21) were infected with *Rickettsia* sp. alone, 1 case (4.76%, 1/21) was co-infected with *Rickettsia* sp. and *Ehrlichia* sp., and 1 case (4.76%, 1/21) was co-infected with *Rickettsia* sp. and *Babesia* sp.

## Discussion

Among the 21 Malayan pangolins seized by customs, 80.95% (17/21) were parasitized by at least one tick, which is much higher than previous reports on Malayan pangolins [9] and Formosan pangolins [12]. This high infection rate may be related to mutual transmission among pangolins during the smuggling process. When seized, all pangolins were in an unhealthy state. Pangolins require specialized diets and feed only on ants and termites. Furthermore, they also show adverse stress responses when stimulated, resulting in immune, respiratory, and digestive system abnormalities. The poor environments, food deficiencies, and stress factors experienced during smuggling caused physical deterioration, providing an ideal breeding ground for ticks and pathogenic transmission.

As early as the 1950s, Kohls (1957) reported that Sunda pangolins, wild boars, bats, hyenas, bears, sambar, water monitors, long-tailed skinks, and hill turtles could be infected with *A*. *javanense* ticks. Subsequent research showed that *A*. *javanense* is the most common tick in Asian, Indian, Sunda, Malayan, and Chinese pangolins and can infect humans and transmit zoonotic pathogens [9]. In our research, ticks collected from the confiscated Malayan pangolins were identified as *A*. *javanense*. After ruling out viral infection, we tested for bacteria that may be carried by *A*. *javanense*, including *Rickettsia*, *Anaplasma*, *Ehrlichia*, *Babesia*, *Theileria*, and *Hepatozoon*.

*Rickettsiaceae* are a diverse group of obligatory intracellular gram-negative bacteria and include the *Rickettsia*, *Anaplasma*, *Ehrlichia*, *Orientia*, and *Coxiella* genera [20], which is a neglected pathogen mainly transmitted by ticks, and its infection status in animals, especially in wild animals, is not yet clear. Moreover, human rickettsial diseases are mostly endemic in nature and under-diagnosed in developing countries such as India [21], and underdeveloped countries in Asian and Africa [22]. Pangolins are wild animals that is difficult to raise on a large scale artificially, which poses a significant obstacle to the study of pathogens that can infect pangolins and cause them to become diseased or even die. Therefore, there are no explicit reported about the severity or even death of pangolin infections caused by pathogens including *Rickettsiaceae*. The *Rickettsia* genus is divided into the typhus group (TG) and spotted fever group (SFG), while *Orientia tsutsugamushi* and *Orientia chuto* belong to the scrub typhus group (STG) [23]. Common clinical symptoms of spotted fever include anorexia, fever, rash, cutaneous ulcers, coma, convulsions, and other neurological symptoms [24]. The symptoms and lesions of the confiscated pangolins were highly consistent with the above description of spotted fever.

In the present study, *Rickettsia* had the highest positive rate in tick samples and pangolin samples (90.91% and 42.86%, respectively). Phylogenetic analysis was performed using the *Rickettsiaceae* 17 kDa gene. Results showed that more than half of all *Rickettsia* spp. found in the tick and all the *Rickettsia* spp. found in pangolin samples closely resembled spotted fever group (SFG) members, *Candidatus Rickettsia* and *Rickettsia rhipicephali* (GenBank MH932038, MH932031, and CP003342). The confiscated pangolins, already suffering from malnutrition and weakened immunity, were highly likely to accelerated in their deaths by spotted fever caused by *Rickettsia* spp. from *A*. *javanense*.

We also found great differences between the *Rickettsiaceae* 17 kDa genes amplified from different ticks on the same pangolin. Of note, of the six tick samples collected from Pangolin No. 16, the similarity between sequences was 76.30%–100.00%, whereas the similarity between the PT5-1 and PT5-2 tick samples was only 62.10%. These results not only suggest that *Rickettsia* infection may be the leading cause of death in the pangolins, but also imply that pangolin smuggling may create ideal conditions for the transmission and recombination of *Rickettsia* spp..

The tick samples positive rates of *Anaplasma* spp. and *Ehrlichia* spp. were 6.06%, respectively, and *Ehrlichia* sp. was only positive in Pangolin12. Phylogenetic analysis of the 16S rRNA genes showed that the two *Anaplasma* spp. were most closely related to the uncultured *Anaplasma* sp. (GenBank KU189193) (99.30% and 99.65%) and formed a clade with *Anaplasma bovis*. The *Ehrlichia* spp. obtained from pangolin and tick samples exhibited 99.46%, 99.65%, and 99.64% similarity, respectively, with *E*. *ruminantium* (GenBank NR_074155.1) (Fig 5), which is considered to be the pathogenic agent of Heartwater [25]. Although the positive rate is very low, tick bites caused *Ehrlichia* transmission in pangolins cannot be ignored.

We also carried out detection of the *Babesia*, *Theileria*, and *Hepatozoon* genera, which live in mammalian blood cells and can cause potentially fatal diseases in infected animals [26]. *Babesia* spp. were detected in both tick and pangolin samples, with positive rates of 15.15% and 4.76%, respectively, while *Theileria* sp. and *Hepatozoon* sp. were not detected. The clinical symptoms observed in some pangolins partially coincided with symptoms of *Babesia* infection, such as anorexia, hematuria, and dyspnea [27]. Thus, we hypothesized that *Babesia* infection was not the primary cause, not rule out it might accelerate death of a few pangolins.

Of note, we also detected *Colpodella* spp. in the tick samples, which are close relatives of the phylum Apicomplexa, including *Babesia* and *Plasmodium* [28]. While earlier research suggested that most *Colpodella* species are free-living and feed on protists [29], subsequent studies have identified these species in blood, tick, soil, and fecal samples [30–32]. Studies in China indicate that *Colpodella* may also infect humans and induce neurological symptoms [31, 33]. Among the 33 ticks tested in this study, six (overall prevalence 18.18%) were infected with *Colpodella*, with two sequences similar to ticks from Qinghai (GenBank MH012046) and Yunnan (GenBank MH208621), and the other four sequences similar to a *Colpodella* sp. (GenBank KT600661) implicated to cause neurological symptoms upon infection. This study is the first to report on *Colpodella* sp. infection in *A*. *javanense* ticks carried on pangolins. Given the limited research, we cannot currently speculate on the relationship between ticks carrying *Colpodella* sp. and the clinical symptoms or death in the Malayan pangolins. The route of transmission of *Colpodella* is also unknown, but it is likely to be through tick bites [31]. While the public health threat posed by *Colpodella* is not yet known, further research is needed on the role of wild animals that may carry ticks, such as pangolins, in the transmission of this parasite.

## Conclusions

This study suggests that spotted fever caused by *Rickettsia* spp. from *A*. *javanense* accelerates the death of most confiscated unhealthy pangolins. Co-infection of *Rickettsia* spp. with *Ehrlichia* spp., or *Babesia* spp. may have accelerated deterioration and eventual death of a few pangolins. Identification of the *Colpodella* pathogen in *A*. *javanense* ticks carried by the pangolins warrants further study to investigate its transmission and pathogenic features. Furthermore, the diagnosis, treatment, and surveillance of TBDs are needed to improve rescue operations for pangolins and other wild animals, which should also shed light on the prevention and control of zoonotic diseases.
